# Improving Awareness of Safe Sleeping Practices for Babies on a Mother and Baby Psychiatric Unit

**DOI:** 10.1192/j.eurpsy.2022.1933

**Published:** 2022-09-01

**Authors:** I. Puttaroo, K. Sheppard, J. Lewin, S. Sriranjan

**Affiliations:** Central North West London NHS Trust, Coombe Wood Mother And Baby Unit, Park Royal Centre For Mental Health, London, United Kingdom

**Keywords:** Paediatrics, Patient safety, Perinatal psychiatry, SIDS

## Abstract

**Introduction:**

SIDS is the sudden, unexpected and unexplained death of a baby. Safe sleeping practices can help to reduce a baby’s risk of SIDS. At the Coombe Wood Mother & Baby Unit (MBU), it was found that many patients were opting to co-sleep with their babies which contradicts safe sleeping guidelines.

**Objectives:**

To improve patient awareness of the condition SIDS and to implement an interactive training session improving awareness of safe sleeping practices for babies. To improve patients’ confidence in implementing safe sleeping practices for their babies; thus reducing the risk of SIDS occurring.

**Methods:**

The Lullaby Trust™ is a charity that raises awareness of SIDS and provides expert advice on safe sleep for babies. An interactive training session for patients was organised by incorporating published materials from The Lullaby Trust™, facilitated by medical and occupational therapy staff on the MBU. The participants filled out a pre-training and post-training questionnaire to test the effectiveness and quality of the training session.

**Results:**

The participants’ average level of confidence in knowing and applying safe sleeping practices for their babies doubled following the training session (from 2.3→4.8 and 2.6→5 respectively, with 5 meaning “Very Confident.”) The average level of knowledge of SIDS also increased from 1.6→4.4 (with 5 meaning “A Lot” of Knowledge.)

**Conclusions:**

We were surprised at the low level of knowledge and confidence the patients had regarding safe sleeping practices for their babies. This project shows how interactive, ward-based training can be an effective way to engage and stimulate patients into improving the safety of their baby care.

**Disclosure:**

No significant relationships.

